# Tumor-Derived Exosomes Induce the Formation of Neutrophil Extracellular Traps: Implications For The Establishment of Cancer-Associated Thrombosis

**DOI:** 10.1038/s41598-017-06893-7

**Published:** 2017-07-25

**Authors:** Ana C. Leal, Daniella M. Mizurini, Tainá Gomes, Natalia C. Rochael, Elvira M. Saraiva, Marcos S. Dias, Claudio C. Werneck, Micheli S. Sielski, Cristina P. Vicente, Robson Q. Monteiro

**Affiliations:** 1 Institute of Medical Biochemistry Leopoldo de Meis, Federal University of Rio de Janeiro, Rio de Janeiro, Brazil; 2 Department of Immunology, Institute of Microbiology Paulo de Góes, Federal University of Rio de Janeiro, Rio de Janeiro Rio de Janeiro, Brazil; 3 International Research Center, A.C.Camargo Cancer Center, São Paulo, Brazil; 4 Department of Biochemistry and Tissue Biology, Institute of Biology, University of Campinas, São Paulo, Brazil; 5 Department of Structural and Functional Biology, Institute of Biology, University of Campinas, São Paulo, Brazil

## Abstract

Cancer patients are at an increased risk of developing thromboembolic complications. Several mechanisms have been proposed to explain cancer-associated thrombosis including the release of tumor-derived extracellular vesicles and the activation of host vascular cells. It was proposed that neutrophil extracellular traps (NETs) contribute to the prothrombotic phenotype in cancer. In this study, we evaluated the possible cooperation between tumor-derived exosomes and NETs in cancer-associated thrombosis. Female BALB/c mice were orthotopically injected with 4T1 breast cancer cells. The tumor-bearing animals exhibited increased levels of plasma DNA and myeloperoxidase in addition to significantly increased numbers of circulating neutrophils. Mice were subjected to either Rose Bengal/laser-induced venous thrombosis or ferric chloride-induced arterial thrombosis models. The tumor-bearing mice exhibited accelerated thrombus formation in both models compared to tumor-free animals. Treatment with recombinant human DNase 1 reversed the prothrombotic phenotype of tumor-bearing mice in both models. Remarkably, 4T1-derived exosomes induced NET formation in neutrophils from mice treated with granulocyte colony-stimulating factor (G-CSF). In addition, tumor-derived exosomes interacted with NETs under static conditions. Accordingly, the intravenous administration of 4T1-derived exosomes into G-CSF-treated mice significantly accelerated venous thrombosis *in vivo*. Taken together, our observations suggest that tumor-derived exosomes and neutrophils may act cooperatively in the establishment of cancer-associated thrombosis.

## Introduction

The correlation between malignancy and procoagulant states has long been established^1, 2^. The occurrence of cancer is commonly associated with the manifestation of a variety of clinical thrombotic syndromes including local and systemic venous and arterial thromboses^3, 4^. In fact, thrombosis is frequently associated with a worse prognosis of neoplastic disease as it is often diagnosed as the first clinical manifestation of a tumor. Despite the large number of epidemiological studies investigating the incidence, prevalence and treatment of thrombosis in cancer patients, the mechanisms underlying the pathogenesis of this process have not been completely elucidated.

The release of extracellular vesicles (EVs) by tumor cells has been associated with several pro-tumoral processes including primary growth, angiogenesis, metastasis, and modulation of the tumor microenvironment and host immune response^5, 6^. Two main types of EVs have been identified in cancer patients’ biological fluids: microvesicles are relatively large size (0.1–1 μm diameter) membrane fragments shed from the cell surface and exosomes, which are composed of particles preformed within multivesicular bodies that fuse with the plasma membrane to release vesicles between 50 and 200 nm^5, 6^. Several studies have correlated the increased plasma levels of tumor-derived tissue factor (TF)-positive microvesicles with a prothrombotic state in both animal models^7–9^ and patients^10^. However, this correlation has not been observed in a number of cancer types including gastric, colorectal, brain and breast cancer^11–13^. From these data, two explanations have been proposed: 1) TF-independent mechanisms may operate to favor cancer-associated thrombosis; 2) EVs in the nanoscale range (exosomes), which have not been evaluated in most of the thrombosis studies, may have an underestimated role.

Increased leukocyte count is a well-known predictor of cancer-associated thrombosis^14, 15^. In fact, recent studies have shown that neutrophils play an important role in arterial^16^ and venous^17^ thrombus development. The involvement of neutrophils in thrombus formation is dependent on the formation of neutrophil extracellular traps (NETs)^18, 19^, which are described as web-like structures of DNA and proteins formed through a process called NETosis^20, 21^. The negatively charged surface of NETs results in the activation of contact phase proteins such as FXII. In addition, NETs activate platelets and inactivate local endogenous anticoagulants^22, 23^. Therefore, treatment with DNase I, which degrades NETs, severely impairs the development of deep vein thrombosis in mice^18, 19^.

NETs have been proposed as important players in cancer-associated thrombosis^15, 22–24^. Demers and colleagues showed that neutrophilic mouse models of cancer exhibit systemic markers of NET formation and neutrophils that are more prone to NET formation *ex vivo* ^23, 25^. Tumor-associated neutrophilia and propensity to NET formation seems to be directed correlated with increased levels of granulocyte colony-stimulating factor (G-CSF)^23–25^. These tumor models also exhibit spontaneous thrombus formation. Remarkably, the prothrombotic state could be reproduced by treating tumor-free mice with G-CSF combined with low doses of LPS^23^.

In the present study, we employed a murine breast cancer model to address the possible cooperation between tumor-derived exosomes and neutrophils in the establishment of cancer-associated thrombosis. Our results demonstrate that NET formation is crucial for the acceleration of venous and arterial thrombus formation in tumor-bearing mice. Remarkably, tumor cell-derived exosomes induce NET formation in neutrophils from G-CSF-treated mice and accelerate venous thrombus formation in tumor-free neutrophilic mice. Our observations suggest that tumor-derived exosomes and neutrophils act cooperatively in the establishment of cancer-associated thrombosis.

## Results

### Mice bearing 4T1 tumors exhibit a NET-dependent prothrombotic state

**Figure 1:**
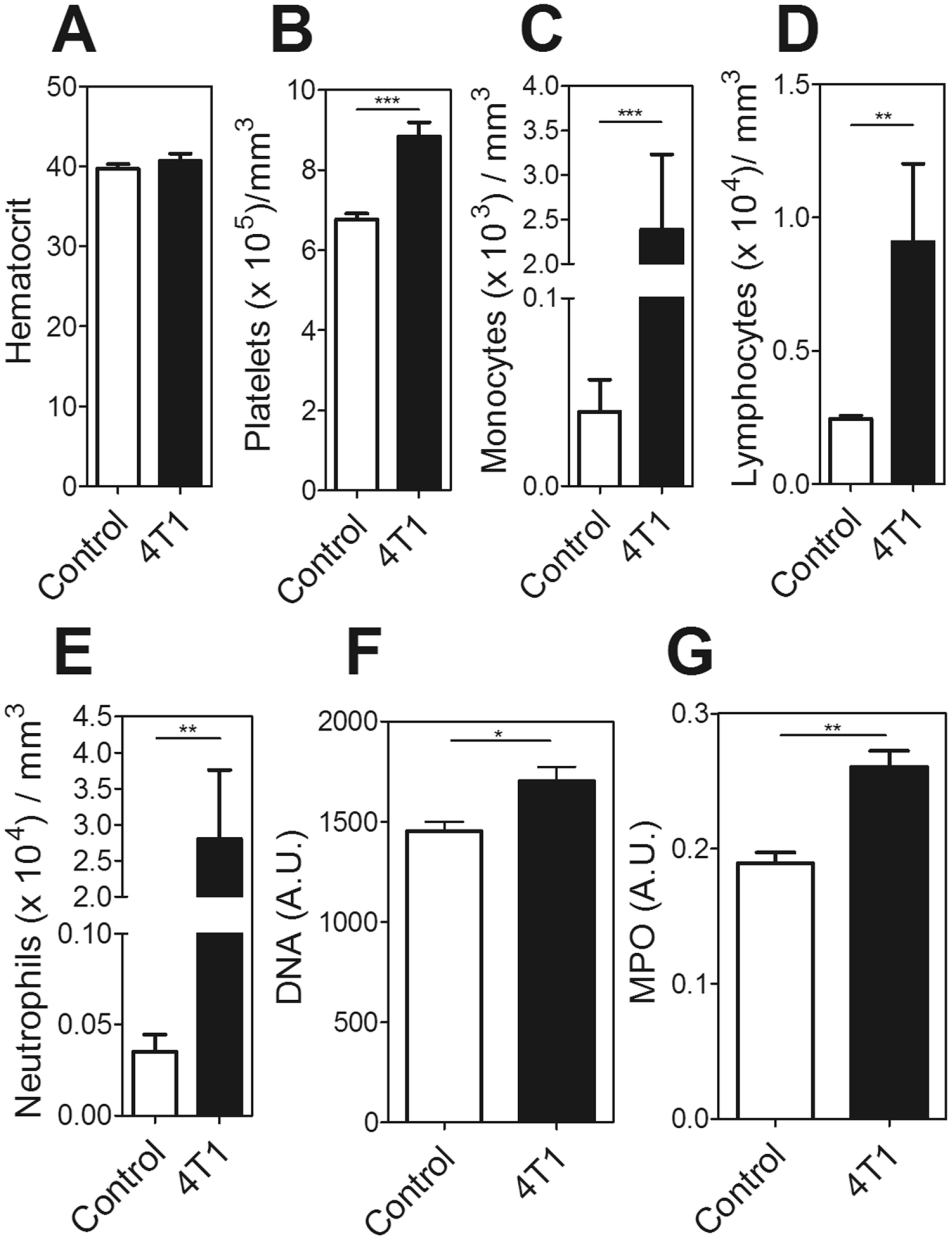
Tumor-bearing mice display systemic signs of neutrophil activation. 4T1 tumor cells (5 × 10^4^) were orthotopically injected into the mammary fat pads of female BALB/c mice. Blood was collected for hemogram analysis 21 days after tumor cell inoculation as described in the Methods section. The following parameters were analyzed: (**A**) hematocrit; (**B**) platelet count; (**C**) monocyte count; (**D**) lymphocyte count and (**E**) neutrophil count in control (open bars) and 4T1 tumor-bearing mice (black bars). The plasma levels of (**F**) DNA and (**G**) myeloperoxidase (MPO) were evaluated in control (open bars) and 4T1-bearing mice (black bars) as described in the Methods section. *P < 0.05; **P < 0.01; unpaired, two-tailed Student’s t-test. N = 5 mice per group.

The 4T1 murine mammary carcinoma cell line is a highy aggressive and metastatic cell line whose orthotopic inoculation into female BALB/c mice induces the rapid growth of solid tumor masses^26^. Moreover, 4T1 tumors have been described as potent G-CSF producers^23, 27^, resulting in the dramatic elevation of circulating neutrophils. Here, we observed increased platelet, monocyte, lymphocyte and neutrophil counts in mice bearing 4T1 tumors, compared to control mice, 3 weeks after inoculation (Fig. 1A–E). The tumor-bearing mice displayed systemic signs of neutrophil activation and NET induction, as indicated by increased plasma levels of extracellular DNA (Fig. 1F) and myeloperoxidase (Fig. 1G).

**Figure 2:**
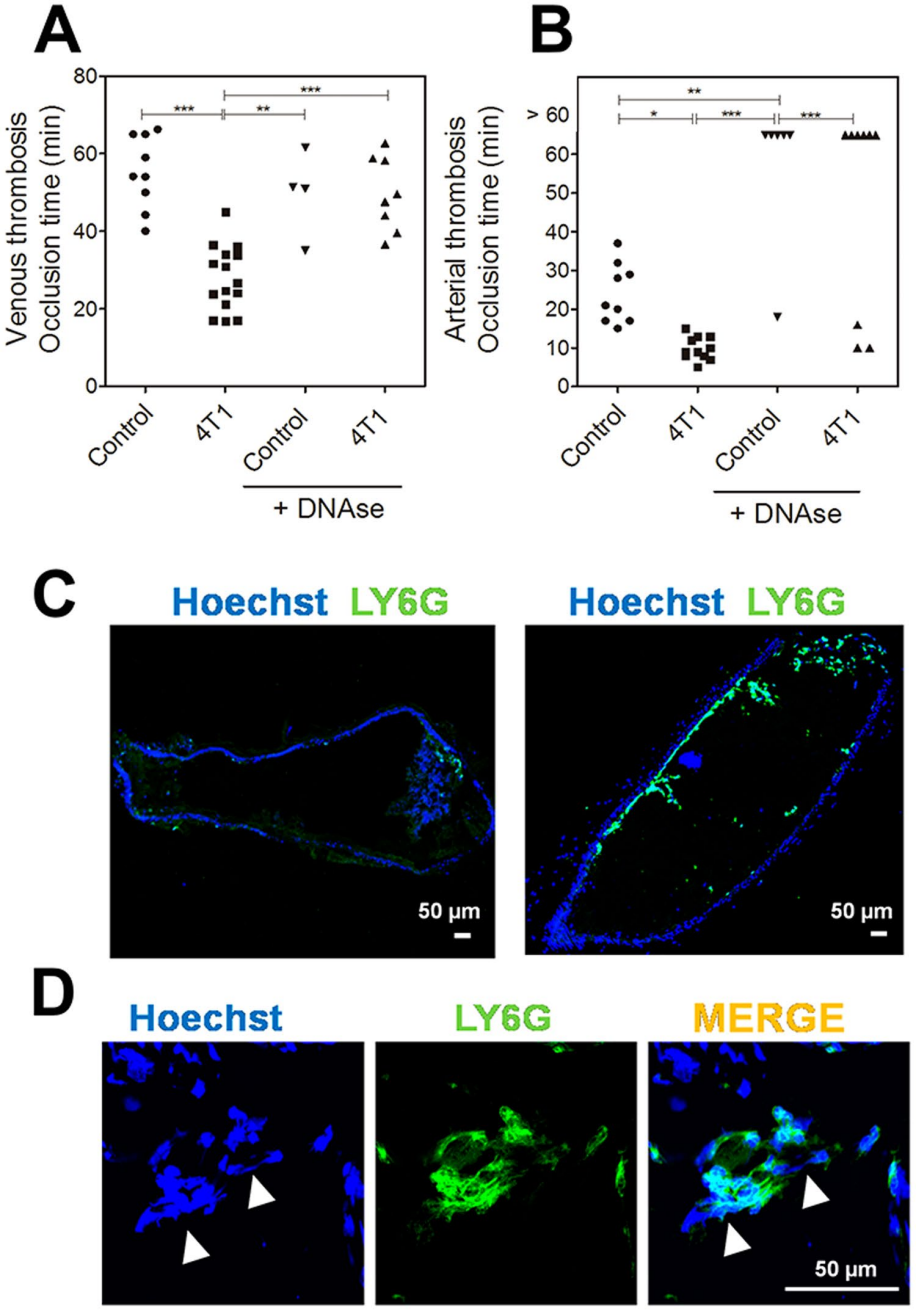
4T1 tumor-bearing mice exhibit a NET-dependent prothrombotic state. (**A**) Venous thrombosis was evaluated in the Rose Bengal/laser-induced injury in control (●, n = 9) and 4T1 tumor-bearing (■, n = 15) mice as described in the Methods section. Alternatively, control (▼, n = 4) and 4T1-bearing (▲, n = 8) mice were treated with DNase 1 (10 μg, i.v.) 15 min before vessel injury. The data represent the occlusion time after photochemical injury in the jugular vein. (**B**) Arterial thrombosis was evaluated in the ferric chloride model in (●, n = 9) control and (■, n = 9) 4T1 tumor-bearing mice as described in the Materials and Methods section. Alternatively, control (▼, n = 6) and 4T1-bearing (▲, n = 9) mice were treated with DNase 1 (10 μg, i.v.) 15 min before vessel injury. The data represent the occlusion time after ferric chloride injury in the carotid artery. Each data point represents one individual mouse. **P < 0.01; ***P < 0.001; analysis of variance (ANOVA) with Tukey’s posttest. (**C**) Fluorescence microscopy analysis of longitudinal cryosections of arterial thrombi from control (left) and 4T1 tumor-bearing mice (right). (**D**) Higher magnification of the thrombus from a 4T1 tumor-bearing mouse. The sections were stained with anti-Ly6G (green) and Hoechst (blue). Extracellular DNA fibers are indicated by arrows. Bars = 50 μm.

The systemic signs of neutrophil activation in 4T1-bearing mice were previously associated with microthrombosis, which coincides with the appearance of NETs^23^. Here, we evaluated the involvement of NETs in tumor-associated venous or arterial thrombosis. To address this question, we first employed a venous thrombosis model in which thrombus formation was induced by Rose Bengal/laser photochemical injury in the jugular vein. Figure 2A shows that the occlusion times were significantly lower in tumor-bearing mice than in controls (27.9 ± 2.2 min *vs*. 56.7 ± 3.2 min). Next, mice were subjected to a model of arterial thrombosis in which thrombus formation was triggered in the carotid artery by injury with ferric chloride. As seen in the venous model, 4T1-bearing mice exhibited a significant reduction in occlusion time in comparison to control animals (9.8 ± 0.9 min *vs*. 32.7 ± 6.6 min) (Fig. 2B). To determine the participation of NETs in the prothrombotic state, mice were treated with DNase 1 prior to thrombosis induction. Remarkably, in the venous thrombosis model, the occlusion times of control and tumor-bearing mice were very similar upon treatment with DNase 1 (49.7 ± 5.4 min *vs*. 49.6 ± 3.3 min) (Fig. 2A). Interestingly, treatment with DNase 1 abolished arterial thrombus formation in most of the animals regardless of the presence of 4T1 tumors, suggesting that NETs are essential for thrombus formation upon ferric chloride-induced vessel injury. Arterial thrombi were further analyzed by immunofluorescence. As expected, thrombi from 4T1-bearing mice were highly enriched in cells that stained for Ly6G, a well-recognized marker for peripheral neutrophils^28^, as compared to thrombi from control mice (Fig. 2C). In addition, a strong co-localization of Ly6G with extracellular fiber-like DNA was observed, indicating the presence of NETs (Fig. 2D).

### Tumor-derived exosomes induce NET release

**Figure 3:**
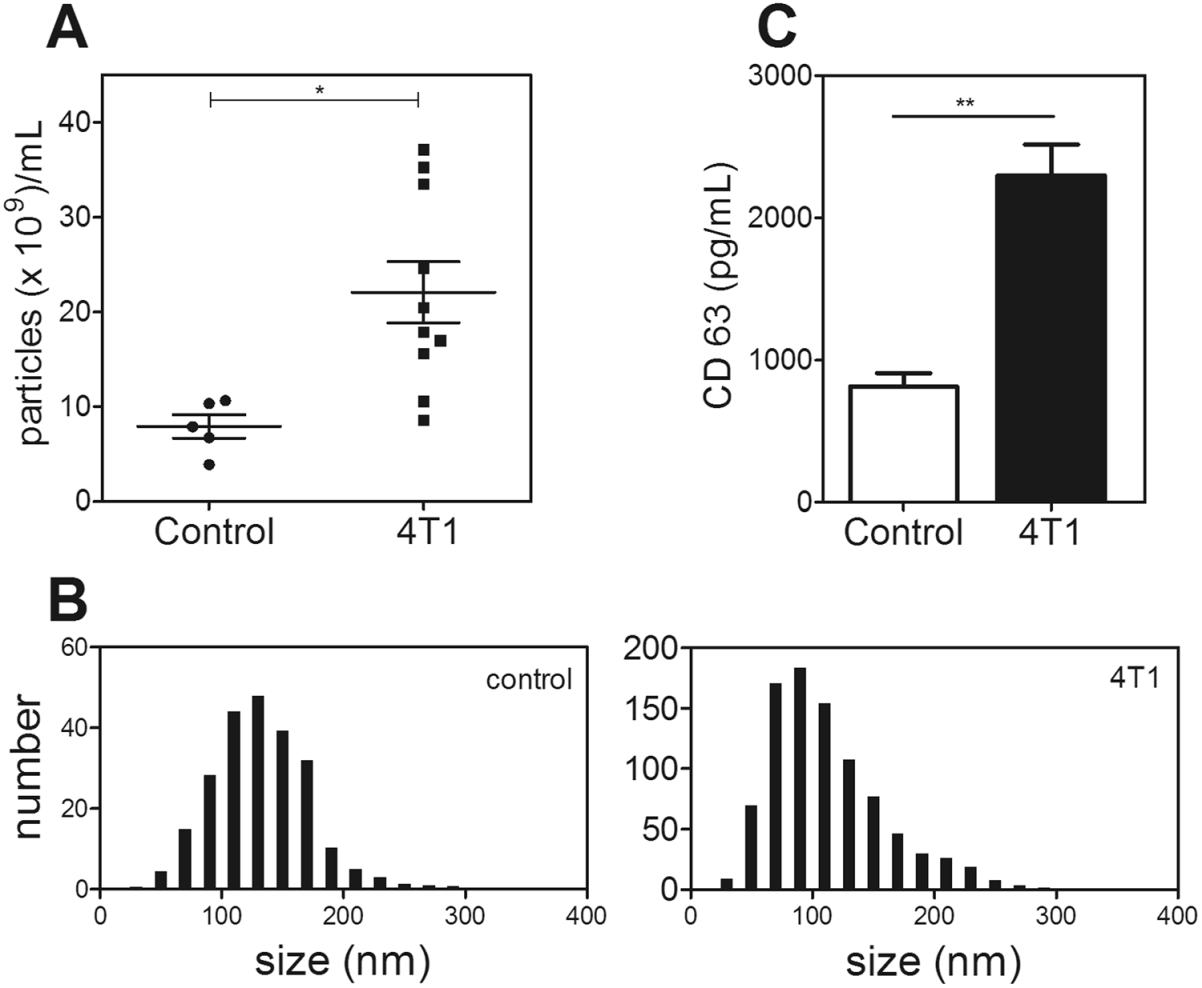
Exosome levels are increased in tumor-bearing mice. (**A**) Quantification of exosomes isolated from the plasma of control (●, n = 5) and 4T1 tumor-bearing (■, n = 10) mice. (**B**) Representative histogram indicating the size distribution of exosomes isolated from control (left panel) and 4T1 bearing-mice (right panel). Exosomes were isolated from the plasma as described in the Methods section. (**C**) CD63 was quantified in the plasma of control (open bar) and 4T1-bearing mice (black bar) as described in the Methods section. *P < 0.05; **P < 0.01; unpaired, two-tailed Student’s t-test.

Several studies have demonstrated that tumor-derived EVs (microvesicles and exosomes) modulate host immune cells and thus contribute to cancer progression^5, 29^. Quantitative analysis of EVs in plasma revealed a significantly higher number of particles in tumor-bearing mice than in controls (Fig. 3A). Nanotracking analysis showed that most of the particles are in the 80–110 nm range, suggesting the accumulation of exosome-like vesicles (Fig. 3B). These data were confirmed by the increased levels of CD63, a known exosome marker^30^, in the plasma of 4T1-bearing mice in comparison to controls (Fig. 3C).

**Figure 4:**
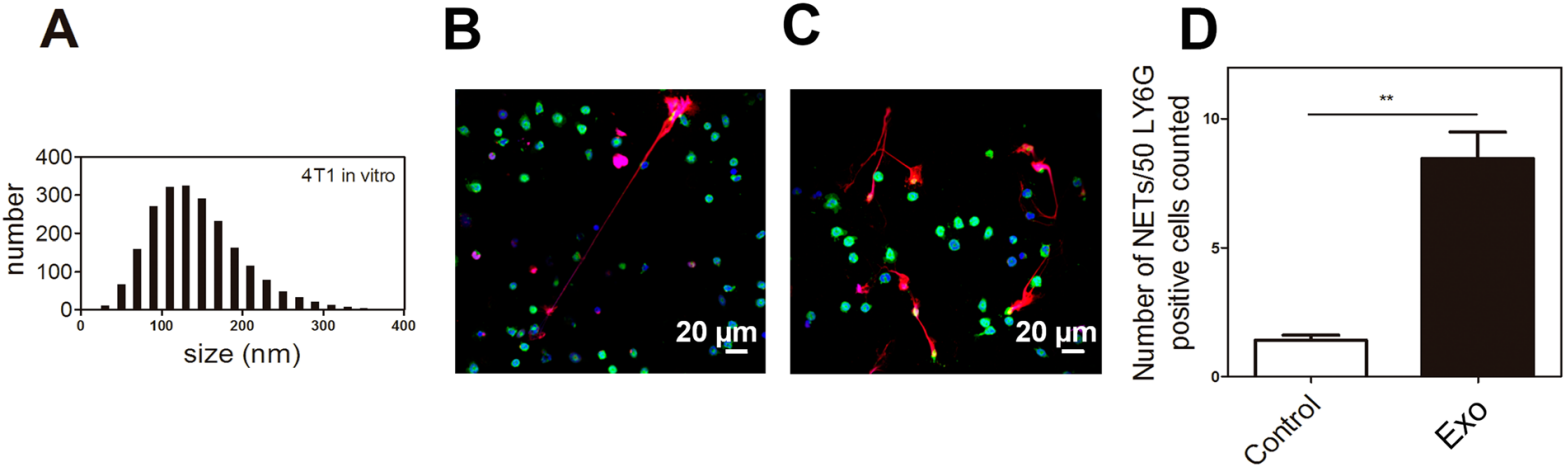
Tumor-derived exosomes induce NET formation. (**A**) Histogram indicating the size distribution of exosomes isolated from the culture supernatants of 4T1 cells. Exosomes were isolated and quantified as described in the Methods section. Bone marrow cells isolated from G-CSF-treated mice were incubated for 3 h at 37 °C in the absence (**B**) or presence (**C**) of 0.1 µg of 4T1-derived exosomes (Exo). Immunofluorescence staining for DNA (blue), Ly6G (green) and citrullinated histone (red) was performed as described in the Methods section. Scale bars = 20 μm. (**D**) The quantification of NETs was performed as described in the Methods section. **P < 0.01; unpaired, two-tailed Student’s t-test. Experiments were run in triplicate (n = 3).

To evaluate whether tumor-derived exosomes could influence NET release, we harvested exosomes from cultured 4T1 cells and evaluated their effect on isolated mouse bone marrow cells. Most of the 4T1-derived exosomes were within the same size range (100–120 nm) of the particles observed in the plasma of tumor bearing-mice (Fig. 4A). The increased predisposition of tumor-bearing mice for NET formation has been attributed at least in part to the increased G-CSF levels in these mice. In this context, we treated bone marrow cells from G-CSF-treated mice with 4T1-derived-exosomes. We noticed a significant increase in the amount of extracellular filamentous DNA derived from Ly6G^+^ cells in the cells treated with exosomes (Fig. 4C) compared to the untreated bone marrow cells (Fig. 4B). The extracellular fibers were positive for citrullinated histone staining, thus confirming that exosomes derived from tumor cells induce NET formation by G-CSF-primed neutrophils (Fig. 4D).

### NETs serves as scaffold for tumor-derived procoagulant exosomes

**Figure 5:**
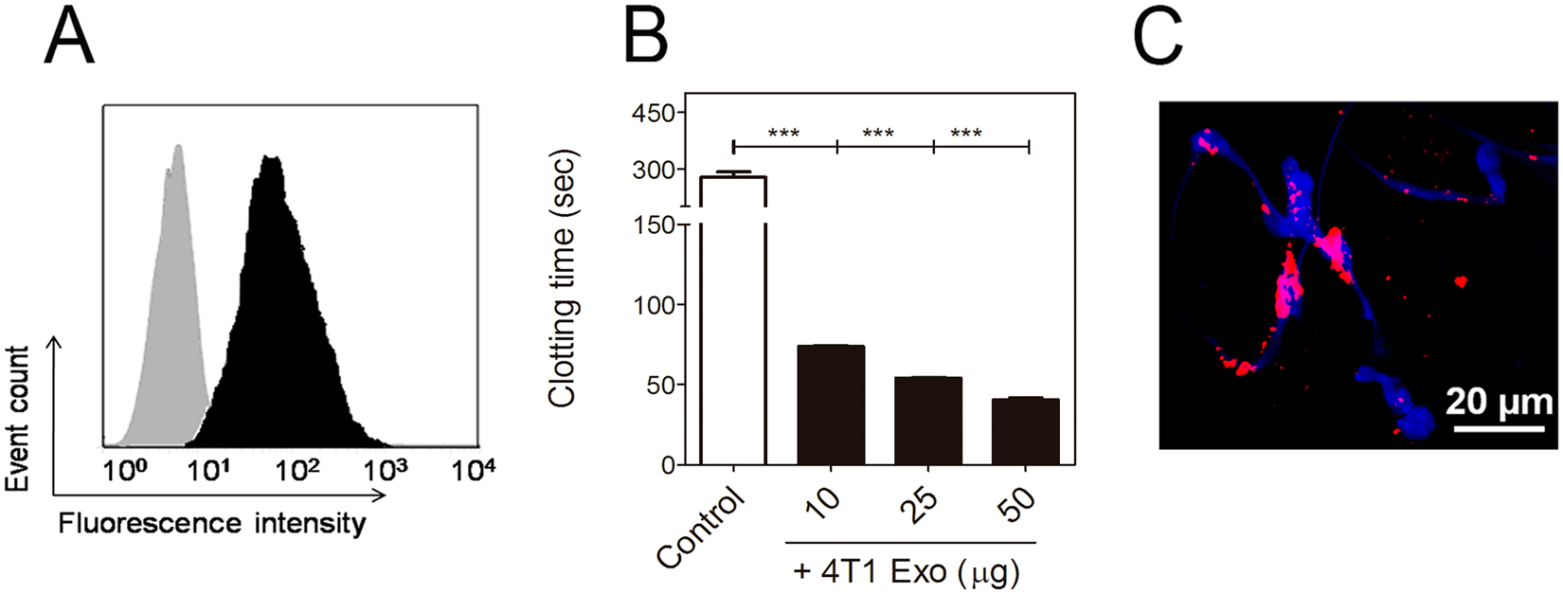
NETs interact with tumor-derived procoagulant exosomes. (**A**) Flow-cytometric analysis of TF expression in 4T1 cells. Black region represents labeling with a rabbit polyclonal anti-murine TF antibody and a phycoerythrin-conjugated secondary antibody. Gray regions represent cells labeled with IgG isotype control and the same phycoerythrin-conjugated secondary antibody. (**B**) Procoagulant activity of 4T1-derived exosomes. Exosomes were isolated and quantified from culture supernatants and further assayed for procoagulant activity, as described in the Methods section. Control bar represents the coagulation time of murine plasma alone. The asterisks indicate P < 0.001 relative to control plasma (Student’s t-test). Experiments were performed in triplicate. (**C**) Representative image showing 4T1-derived exosomes interacting with NETs. Exosomes were labeled with DilC18 (red), and NET DNA was stained with Hoechst 33342 (blue). Cells were isolated and stimulated with PMA for 3 hs to induce NET formation, before incubation with 4T1 exosomes. Scale bar = 20 μm.

The procoagulant properties of tumor cells have been mainly attributed to expression of the clotting initiator protein, tissue factor (TF)^31, 32^. In this context, flow-cytometric analyses revealed high levels of TF antigen on the surface of 4T1 cells (Fig. 5A). Several studies demonstrate that the presence of TF in EVs is strongly dictated by the TF expression status of their cells of origin^33, 34^. Thus, we further evaluated the procoagulant properties of 4T1-derived exosomes. Figure 5B shows that exosomes exhibit a dose-dependent procoagulant effect. The recruitment of procoagulant EVs into the growing thrombus has been proposed as important event during thrombus developmet^35, 36^. At this point, we observed that 4T1 cell-derived exosomes can adhere to NETs *in vitro* under static conditions (Fig. 5C). These results suggest that NETs may recruit tumor-derived exosomes thus contributing to the prothrombotic state in 4T1-bearing mice.

### Treatment with G-CSF and exosomes recapitulates the prothrombotic state of tumor-bearing mice

**Figure 6:**
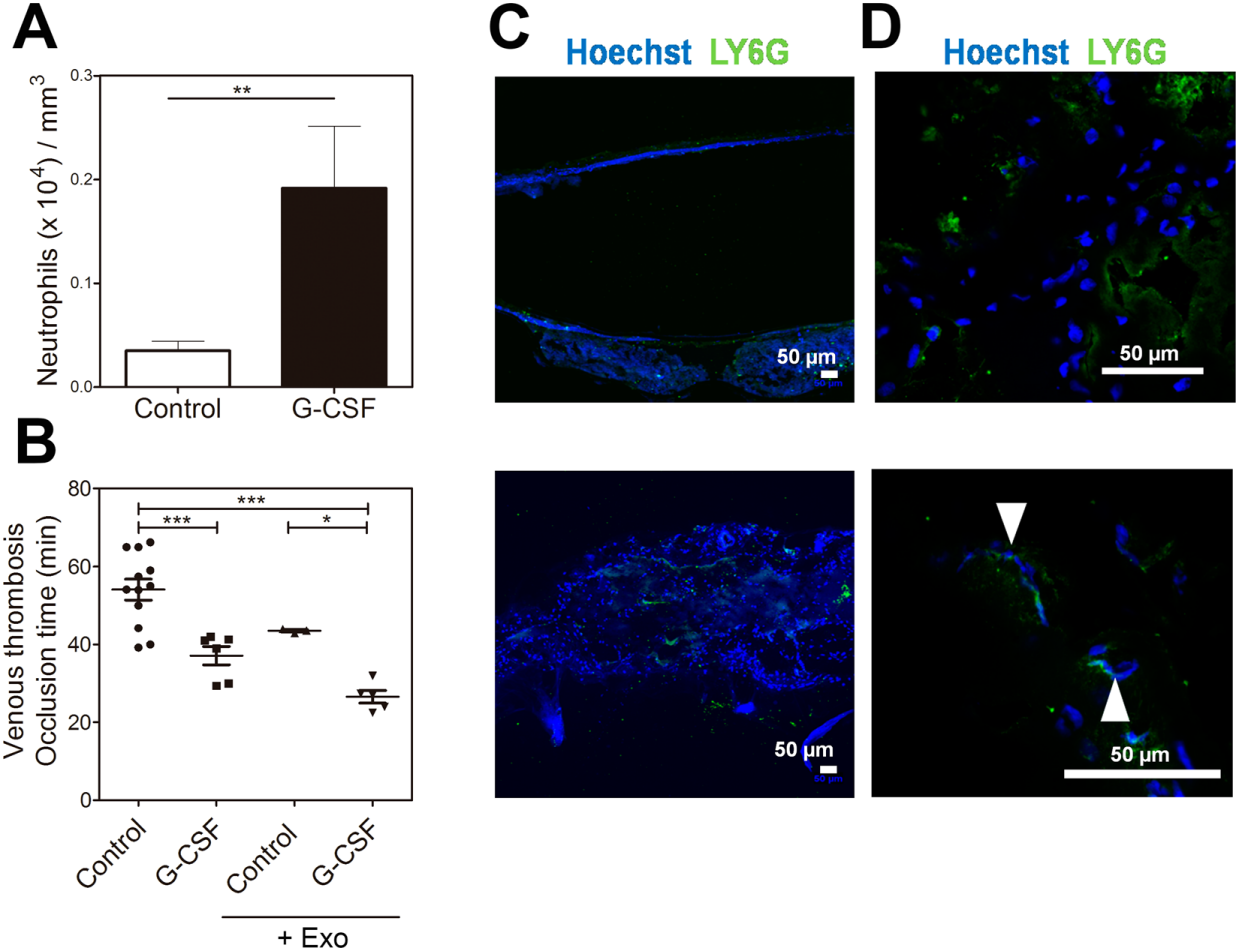
G-CSF and tumor-derived exosomes accelerate venous thrombosis in tumor-free mice. (**A**) Neutrophil counts in the peripheral blood of control (n = 5) and G-CSF-treated (n = 5) mice. (**B**) The venous thrombosis model was applied to control mice (●, n = 12), mice treated with G-CSF (■, n = 6), control mice infused with 100 µg of 4T1-derived exosomes (▲, n = 3), or mice treated with G-CSF infused with 100 µg of 4T1-derived exosomes (▼, n = 5). The data represent the mean occlusion time after photochemical-induced vascular injury, with each data point representing one individual mouse. **P < 0.01; ***P < 0.001; analysis of variance (ANOVA) with Tukey’s posttest. (**C**) Fluorescence microscopy analysis of cryosections of venous thrombi from control mice treated with exosomes (upper) and mice treated with G-CSF + exosomes (bottom). (**D**) Higher magnification of the venous thrombi from control mice treated with exosomes (upper) and mice treated with G-CSF + exosomes (bottom). The sections were stained with Ly6G (green) and Hoechst (blue). Extracellular DNA fibers are indicated by arrows. Bars = 50 μm.

To evaluate the effect of G-CSF-induced neutrophilia and tumor-derived exosomes in the mouse prothrombotic state, we employed the venous thrombosis model in tumor-free mice. Treatment with G-CSF efficiently increased the number of peripheral neutrophils, as shown in Fig. 6A. Neutrophilia induced by G-CSF was sufficient for reducing the occlusion times compared to naïve mice (37.1 ± 2.3 min *vs*. 54.0 ± 2.6 min), as shown in Fig. 6B. Less significant changes were observed upon intravenous administration of 4T1-derived exosomes in G-CSF-untreated mice (43.5 ± 0.3 min). Remarkably, the occlusion times observed in G-CSF-treated mice, which were further infused with 4T1-derived exosomes (26.6 ± 1.6 min), were similar to those recorded for tumor-bearing mice (27.9 ± 2.2 min). Further immunohistochemical analysis confirmed the increased presence of Ly6G + cells and DNA fibers in thrombi from G-CSF/exosomes-treated mice (Fig. 6C).

## Discussion

Several studies have demonstrated that neutrophils actively participate in both arterial and venous thrombus formation. There is strong evidence that most of the prothrombotic properties of neutrophils derive from the formation of neutrophil extracellular traps (NETs). *In vitro* and *in vivo* studies have shown that NETs display several pro-hemostatic effects including the stimulation of platelet aggregation, activation of contact pathway, and degradation of natural coagulation inhibitors^37, 38^. Remarkably, Demers and colleagues^23, 25^ demonstrated that murine cancer models that rely on significant neutrophilia predispose NET formation through mechanisms that have not been fully elucidated.

Here, we have employed a murine breast cancer model that develops a dramatic increase in leukocyte count, a process that parallels the systemic signs of NET formation. In this study, tumor-bearing mice exhibited accelerated arterial thrombus formation in the ferric chloride model. Importantly, we have employed a mild ferric chloride treatment, which has been described as critical for discriminating specific prothrombotic stimulus such as increased FVIII levels^39^. Treatment with DNase 1 abolished thrombus formation in most of the tumor-bearing mice. In fact, we could not observe thrombus formation in most of the tumor-free mice that were treated with DNase 1 prior to challenge with ferric chloride. Other studies have demonstrated that neutrophils are essential for thrombus formation in a mouse model of laser-induced injury^16, 40^. Moreover, blocking NET formation in lupus and atherosclerosis mouse models delays arterial thrombus formation induced by photochemical injury in the carotid artery^41, 42^. In addition, impaired DNase 1-mediated degradation of NETs was recently associated with the manifestation of acute thrombotic microangiopathies in humans^43^. It remains to be determined whether a similar mechanism operates to enhance cancer-associated thrombosis.

Tumor-bearing mice also exhibited a significant acceleration of venous thrombosis in the Rose Bengal/laser-induced model. Interestingly, tumor-bearing animals treated with DNase 1 exhibited occlusion times that were identical to tumor-free mice. Most remarkably, treatment with DNase 1 produced no effect on tumor-free animals. This is strikingly distinct from a recently described mouse model of deep vein thrombosis based on partial flow restriction, in which treatment with DNase 1 or the depletion of neutrophils prevented thrombus formation in most of the animals^17, 19^. Therefore, the photochemical injury model may be helpful for evaluating the contribution of the innate immune system to cancer-associated thrombosis.

The 4T1 murine breast cancer model is characterized by a dramatic increase in the number of circulating neutrophils, with G-CSF being a major determinant for this acquired phenotype^23, 25, 27^. Treatment with G-CSF in humans has long been associated with neutrophil activation^44^ and, more recently, with elevation of NET formation markers^45, 46^. Herein, we observed a significant acceleration of venous thrombus formation in mice treated with G-CSF, although the extent of acceleration was not as dramatic as observed in tumor-bearing animals. G-CSF involvement in an intricate pro-metastatic mechanism involving neutrophils and gamma delta T cells was recently identified^47^. In addition, Kowanetz and colleagues demonstrated that G-CSF mobilizes neutrophils to premetastatic sites, thus increasing the metastatic potential of a number of murine cell lines, including 4T1 cells^27^. Alternatively, blocking G-CSF significantly reduces the number of spontaneous metastases in this cell model.

The increasing number of circulating extracellular vesicles (EVs) present during cancer progression has been widely documented in humans and animal models^2, 10, 48^. EVs have been linked with several aspects of tumor biology, including their ability to interact and modulate host immune cells^48, 49^. In this study, we observed that 4T1-derived EVs, in the exosome size-range, induce NET formation in neutrophils derived from G-CSF-treated mice. Remarkably, the infusion of tumor-derived exosomes into G-CSF-treated animals accelerated thrombus formation to a degree similar to that observed in tumor-bearing animals. Thomas and colleagues recently demonstrated the ability of NETs to trap tumor-derived microvesicles in a murine pancreatic cancer model^50^. In fact, the infusion of high-TF but not low-TF tumor-derived microvesicles enhanced deep vein thrombosis. In our study, we observed that NETs interact with 4T1-derived exosomes. Therefore, NETs may function as scaffold for tumor-derived EVs, either exosomes or microvesicles, thus enhancing their effects on thrombosis in addition to other biological effects. Tumor-derived exosomes have been involved in pre-metastatic niche formation^49^. In this context, Bobrie *et al*. (2012) demonstrated that exosome secretion by tumors contributes to the recruitment of neutrophils into primary tumors *in vivo* ^51^. Remarkably, downregulating exosome secretion by knocking down the small GTPase Rab27a in tumor cells resulted in decreased primary growth and metastasis in a murine breast cancer model.

**Figure 7:**
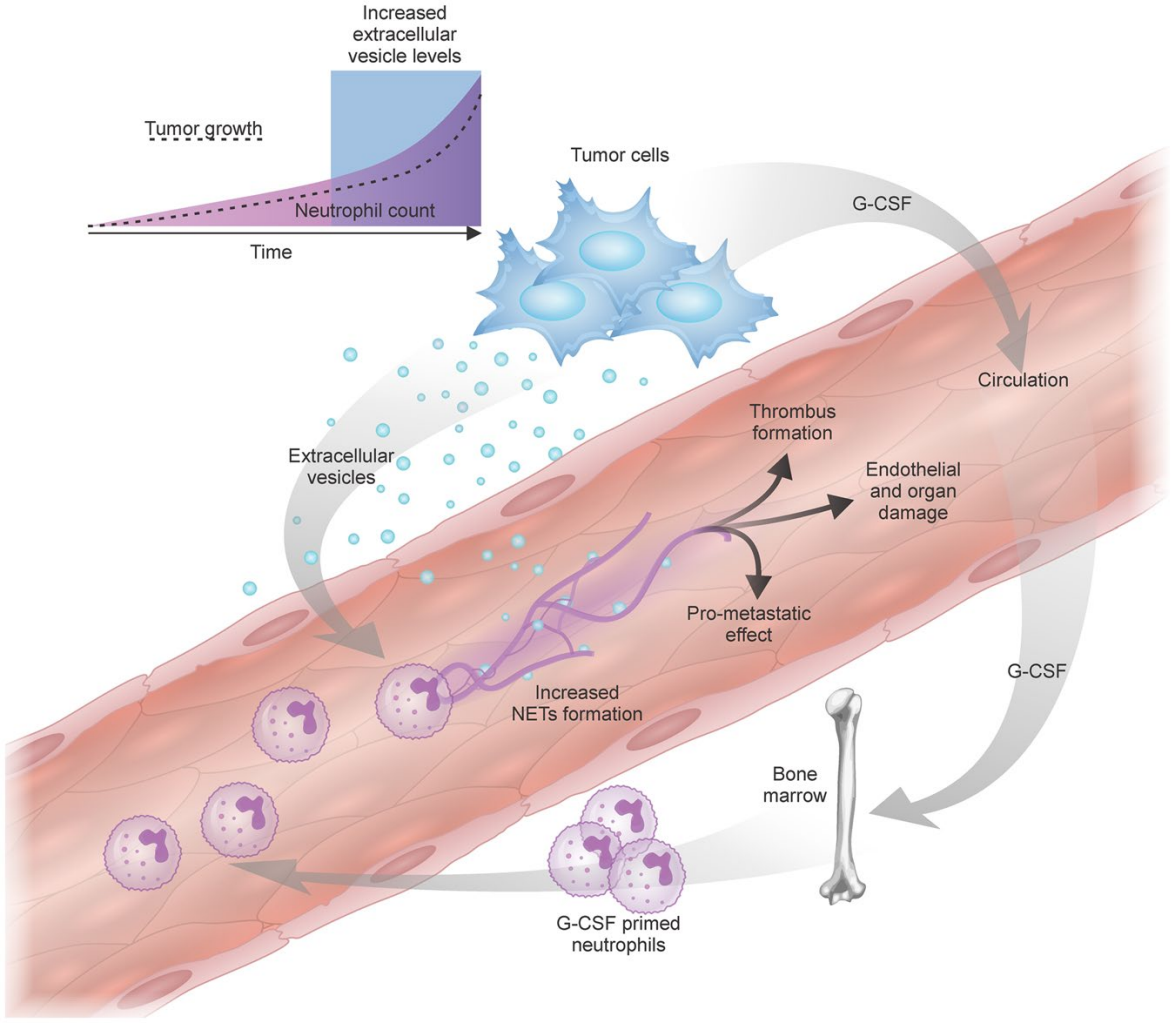
Schematic representation of the cooperation between tumor-derived EVs and neutrophils in tumor progression. The development of a thrombotic state in cancer patients is dependent on tumor-induced systemic alterations, including neutrophilia and increased levels of EVs (microvesicles and/or exosomes). Tumor-derived G-CSF acts in an endocrine fashion, stimulating the bone marrow to produce and export neutrophils to the bloodstream. The tumor also releases a large number of EVs into the circulation. The interaction between tumor-derived exosomes and G-CSF-primed neutrophils favors the release of NETs. Increased NET formation may favor tumor progression in various ways: 1) through the establishment of cancer-associated thrombosis; 2) by facilitating tumor cell arrest and encouraging metastasis; or 3) by promoting vascular damage and organ dysfunction.

Taken together, our results demonstrate that NET formation is crucial for the acceleration of venous and arterial thrombus formation in tumor-bearing mice. 4T1-derived exosomes induce NET formation in neutrophils derived from G-CSF-treated mice and accelerate venous thrombus formation in tumor-free neutrophilic mice. Therefore, we propose that tumor-derived exosomes cooperate with neutrophils in the establishment of cancer-associated thrombosis (Fig. 7). This process might be of particular relevance in tumors that rely on increased G-CSF production and the subsequent neutrophilia that predisposes NET formation. Remarkably, Thålin and colleagues showed that increased G-CSF levels correlates with NET-associated microthrombosis in cancer patients^46^. A parallel increase in tumor-derived EVs may facilitate NET formation both in the tumor microenvironment and systemically, which in turn may impact the occurrence of thrombotic manifestation, primary tumor growth^52^ and metastasis^53^ and vascular damage^54^. Therefore, strategies aimed at preventing NET formation, such as elastase and PAD4 inhibitors^55–57^, may have a significant impact on tumor progression.

## Methods

### Chemicals

AnaSed (xylazine) and Dopalen (ketamine) were purchased from Agribrands (Rio de Janeiro, RJ, Brazil). The Rose Bengal dye (3′,4′,5′,6′-tetrachloro-2,4,5,7-tetraiodofluorescein and Quant-it Picogreen dsDNA were purchased from Thermo Fisher Scientific (Fair Lawn, NJ, USA). Pulmozyme^®^ was from Genentech (San Francisco, CA, USA). The mouse CD63 specific ELISA kit was from Cusabio (Wuhan, Hubei Province, China). SIGMA*FAST*™ OPD, HISTOPAQUE (10771), bovine serum albumim (BSA) and phorbol myristate acetate (PMA) were obtained from Sigma-Aldrich Chemical Co. (St. Louis, MO, USA). ExoQuick-TC™ was from System Biosciences (Mountain View, CA, USA). The rabbit polyclonal antibody against histone H3 (citrulline R2 + R8 + R17; ab5103) was from Abcam (San Francisco, CA, USA) and the goat anti-rabbit IgG labeled with AlexaFluor 488 was from Molecular Probes (São Paulo, SP, Brazil). Hoechst 33342 was purchased from Life Technologies (São Paulo, SP, Brazil). Filgrastim (rh-G-CSF) was purchased from Roche Ltd (Basel, Switzerland).

### Cell culture

The 4T1 cell line, which originated from a spontaneous mammary carcinoma arising in a BALB/c mouse^24^, was purchased from Karmanos Cancer Institute (Detroit, MI, USA). Cells were maintained in high glucose Dulbecco’s modified Eagle medium (DMEM), supplemented with L-GlutaMax, 10 mM sodium carbonate, Hepes Buffer and 10% FCS (fetal calf serum) and maintained at 37 °C in a humidified atmosphere of 5% CO_2_. The cells were kept in culture no more than five passages before inoculation into mice.

### Animals

BALB/c mice were housed under controlled temperature (24 ± 1 °C) and light (12 h light starting at 7:00 a.m.) conditions. All *in vivo* experiments were conducted in accordance with the approved guidelines of the institutional care and use committee from the Federal University of Rio de Janeiro. The animal procedures were approved by the Institutional Committee under protocol number IBQM/089-07/16.

### Tumor induction

Female mice (8–10 wk-old) were inoculated with 5 × 10^4^ 4T1 cells in the fourth mammary fat pad. The animals were monitored every other day for tumor growth, and the tumors were measured with a caliper. Tumor volume was calculated using the formula V = (l × w^2^) × 0.4, where l is the length and w the width.

### Blood cell analysis

The mice were anesthetized as described, and blood was collected into EDTA (1.8 mg/mL) by cardiac puncture. A blood cell count was automatically determined using the CELL-DYN 3500 hematology analyzer (Abbott Diagnostics, Abbott Diagnostics, Illinois, USA).

### Quantification of plasma DNA

Plasma DNA levels were measured using the Quant-it Picogreen dsDNA kit according to the manufacturer’s instructions. Briefly, 5 µL of plasma from control or 4T1 tumor bearing mice and 50 µL of Picogreen (diluted in TRIS buffer) were added per well in an opaque 96-well plate. Fluorescence was measured in an automated spectrofluorimetric reader (Spectra Max Paradigm; Molecular Devices, Menlo Park, CA, USA) at 485 nm excitation and 535 nm emission.

### Quantification of myeloperoxidase and CD63 in plasma

Myeloperoxidase was quantified with a colorimetric assay. Briefly, 50 µL of plasma from control or 4T1 tumor bearing mice was incubated with 25 μL 3,3′,5,5′-tetramethylbenzidine and 50 μL H_2_O_2_. The mixture was incubated for 5 minutes at 37 °C and the reaction was interrupted by the addition of 100 μL H_2_SO_4_ (2 M). The absorbance was determined at 405 nm using a Spectra Max Paradigm spectrophotometer. The level of CD63 in the plasma of control or 4T1 tumor-bearing mice was determined using a mouse CD63 specific ELISA kit according to the manufacturer’s instructions. The results obtained in our experiments were calculated by standard curve interpolation and expressed in pg/mL.

### Venous thrombosis model

Mice were anesthetized as described above. The jugular vein was isolated through a midline cervical incision, and blood flow was continuously monitored using a 0.5 PSB Doppler flow probe coupled to the TS420 flow meter. Rose Bengal dye (75 mg/kg body weight) was diluted to 15 mg/mL in PBS and administered via the lateral tail vein. Just before injection, the jugular vein was transilluminated with a 1.5-mV, 540 nm green laser (#25-LGR-193-249, Melles Griot Inc., Carlsbad, CA) immediately proximal to the Doppler probe at a distance of 6 cm. The occlusion time was calculated as the interval between the injection of Rose Bengal dye and complete and stable (no flow for at least 3 min) cessation of blood flow. For the experiments with DNase 1, the mice were treated intravenously with 10 µg of Pulmozyme^®^, 15 min prior to the induction of thrombosis. For the experiments with exosomes, the particles were intravenously inoculated into the tail vein 15 min prior to Rose Bengal dye injection.

### Arterial thrombosis model

The evaluation of arterial thrombosis was performed as previously described^58^ with minor modifications. In brief, control or 4T1-tumor bearing BALB/c mice were anesthetized with intramuscular xylazine (16 mg/kg) followed by ketamine (100 mg/kg). The right common carotid artery was isolated through a midline cervical incision, and blood flow was continuously monitored using a 0.5-VB Doppler flow probe coupled to a TS420 flow meter (Transonic Systems, Ithaca, NY, USA). Thrombus formation was induced by applying a piece of filter paper (1 × 2 mm) saturated with 5% FeCl_3_ solution on the adventitial surface of the artery for 2 min. After exposure, the filter paper was removed and the vessel was washed with sterile saline. Carotid blood flow was continuously monitored for 60 min or until complete occlusion (no flow for at least 3 min) occurred. For the experiments with DNase 1, the mice were treated intravenously with 10 µg of Pulmozyme^®^, 15 min prior to the induction of thrombosis.

### Immunohistochemistry

Occluded vessel sections were collected and immersed in Tissue-Tek Optimal Cut Temperature Compound (Sakura Finetek, Torrance, CA). Frozen sections (10 µm) were fixed in 4% paraformaldehyde (PFA), washed with PBS, and then blocked with PBS containing 5% BSA. Samples were further incubated overnight with anti-LYG6 antibody conjugated to AlexaFluor 488 (53-5931-80, eBioscience) and Hoechst 33342. Immunofluorescence images were obtained with a confocal microscope (LEICA DMI 4000 with laser system TCS SPE, Leica, Wetzlar, Germany) and analyzed using Fiji Image J software 1.48 g (NIH, USA).

### Isolation of exosomes from plasma samples and cell culture supernatants

The plasma obtained from control or 4T1 tumor-bearing mice was centrifuged for 1000 *g* for 10 min. The resulting supernatant was further diluted in PBS and ultra-centrifuged (Beckman Coulter Optima LE-8OK, 70 Ti rotor) at 100,000 *g* for 1 h at 4 °C. The samples were washed once with PBS and centrifuged again. The pellet was resuspended in ice cold PBS, filtered with a 0.2 μm filter and kept at −20 °C until analysis. Exosomes were isolated from the cell culture medium using ExoQuick-TC™ according to the manufacturer’s instructions. The supernatant obtained from 4T1 cells cultured for 48 h in serum free media, was centrifuged at 3000 *g* for 15 min to remove debris and then incubated overnight at 4 °C with ExoQuick-TC™. This mixture was sequentially centrifuged for 15 and 5 min at 1500 *g*. The supernatant was removed and the pellet was resuspended in ice cold filtered PBS and stored at −20 °C for no more than one week. Exosome protein content was measured using the Pierce Modified Lowry Protein Assay Kit (Thermo Fisher Scientific) according to manufacturer’s instructions.

### Nanoparticle tracking analysis

Nanoparticle tracking analysis was performed using a NanoSight LM10 microscope (NanoSight Ltd, Salisbury, UK). The samples were diluted in PBS. Five videos of 30 seconds were taken for each sample with the detection threshold set at 15. The size and concentration of the samples were determined after analysis of the recorded videos with NTA software (version 2.3). The blur, minimum track length and minimum expected particle size parameters used for the analyses were set automatically.

### ***In vitro*** NET formation

Bone marrow cells were obtained from untreated mice or mice subcutaneously treated with 2.5 μg of G-CSF for 3 days. The cells were then harvested by flushing the femoral bone. The cell suspension was washed with PBS and resuspended in high glucose DMEM (GIBCO). The cells (1 × 10^5^) were then seeded onto 13 mm cover-slips (Glasscyto) and stimulated with 0.1 µg of 4T1-derived exosomes. After incubation for 3 h at 37 °C, cells were fixed with 500 µL of 4% PFA for 10 min, washed 3 times with PBS and incubated for 10 min with blocking solution (PBS, 10% FBS, 5 mg/ml BSA). The samples were then incubated with rabbit polyclonal anti-histone H3 antibody (diluted 1:50 in blocking solution (ab5103, Abcam) and anti-LYG6 antibody conjugated with AlexaFluor 488 (1:100 dilution, 53-5931-80, eBioscience). After three washes with blocking solution, the slides were incubated with goat anti-rabbit IgG labeled with rhodamine (1:500 dilution) (31670, ThermoFisher Scientific) and Hoechst 33342 at 1:1000 for 2 h. Images were captured with a confocal microscope (LEICA DMI 4000 with laser system TCS SPE) and analyzed using Fiji Image J software. NETs were identified on the digitalized images as Hoechst-positive fibrils emanating from cells, with an overall length at least twice as long as the cell diameter, and were counted in at least five fields of view per treatment group. The results were expressed as the number of NETs/50 Ly6G + cells counted.

### Flow-cytometric analyses

For surface detection of TF, 5 × 10^5^ 4T1 cells were resuspended in PBS containing 1% BSA and incubated for 30 min at 4 °C with a rabbit polyclonal antibody against murine TF (200 μg/ml; #4515, American Diagnostica, Stamford, CT, USA). After washing to remove unbound antibody, cells were incubated with a phycoerythrin-conjugated anti-rabbit IgG (Santa Cruz Biotechnology Inc., Santa Cruz, CA, USA). Cells were then washed again and acquired using a BD FACScaliburTM Flow Cytometer (Becton Dickinson). Data were analysed by the BD CellQuest ProTM software (Becton Dickinson).

### ***In vitro*** activation of plasma coagulation

Procoagulant activity of 4T1-derived exosomes was measured by a clotting assay employing normal pooled mouse platelet-poor plasma (PPP). Fifty microliters of exosomes resuspended in PBS at different concentrations were added to 50 μL of PPP containing 3.8% sodium citrate diluted 1:9 (v/v). After 1 min incubation at 37 °C, 100 μL of 25 mM CaCl_2_ was added and the clotting times were recorded on a KC4 Delta Coagulometer (Tcoag Ireland Limited, Wicklow, Ireland).

### Interaction of exosomes with NETs under static conditions

Bone marrow cells were obtained from BALB/C mice as described above. The cells (1 × 10^5^) were then seeded onto 13 mm cover-slips (Glasscyto) and stimulated with 50 nM PMA, a potent inducer of NETosis^20^. After incubation for 3 h at 37 °C, cells were incubated with 1 μg of 4T1-exosomes. Exosomes used in these experiments were previously stained with 3 mM DiIC18 (1,1′-dioctadecyl-3,3,3,3′-tetramethyl-indocarbocyanine perchlorate), a fluorescent lipophilic compound (Invitrogen) that intercalates cell membranes. Cells were further fixed with 500 µL of 4% PFA for 10 min, washed 3 times with PBS and incubated for 10 min with blocking solution (PBS, 10% FBS, 5 mg/ml BSA). The samples were then incubated with Hoechst 33342 at 1:1000 for 2 h. Images were captured with a confocal microscope (LEICA DMI 4000 with laser system TCS SPE) and analyzed using Fiji Image J software.

### Statistical analysis

The results were expressed as the mean ± SEM or were depicted as scatter plots. Statistical analyses were performed using GraphPad Prism 5 (GraphPad Software). One-way analysis of variance (ANOVA) followed by Tukey’s post test was used for comparisons between the test groups. If only two groups were compared, an unpaired 2-tailed Student’s *t-*test was applied. Differences were considered significant when P < 0.05.
